# Predicting the behavioral intentions of hospice and palliative care providers from real-world data using supervised learning: A cross-sectional survey study

**DOI:** 10.3389/fpubh.2022.927874

**Published:** 2022-09-30

**Authors:** Tianshu Chu, Huiwen Zhang, Yifan Xu, Xiaohan Teng, Limei Jing

**Affiliations:** School of Public Health, Shanghai University of Traditional Chinese Medicine, Shanghai, China

**Keywords:** hospice and palliative care, behavioral intention, machine learning, random forest classifier, healthcare providers, cross-sectional study

## Abstract

**Background:**

Hospice and palliative care (HPC) aims to improve end-of-life quality and has received much more attention through the lens of an aging population in the midst of the coronavirus disease pandemic. However, several barriers remain in China due to a lack of professional HPC providers with positive behavioral intentions. Therefore, we conducted an original study introducing machine learning to explore individual behavioral intentions and detect factors of enablers of, and barriers to, excavating potential human resources and improving HPC accessibility.

**Methods:**

A cross-sectional study was designed to investigate healthcare providers' behavioral intentions, knowledge, attitudes, and practices in hospice care (KAPHC) with an indigenized KAPHC scale. Binary Logistic Regression and Random Forest Classifier (RFC) were performed to model impacting and predict individual behavioral intentions.

**Results:**

The RFC showed high sensitivity (accuracy = 0.75; F1 score = 0.84; recall = 0.94). Attitude could directly or indirectly improve work enthusiasm and is the most efficient approach to reveal behavioral intentions. Continuous practice could also improve individual confidence and willingness to provide HPC. In addition, scientific knowledge and related skills were the foundation of implementing HPC.

**Conclusion:**

Individual behavioral intention is crucial for improving HPC accessibility, particularly at the initial stage. A well-trained RFC can help estimate individual behavioral intentions to organize a productive team and promote additional policies.

## Introduction

An aging population is a great challenge for healthcare systems, especially for hospice and palliative care (HPC) aiming to improve end-of-life quality. The number of people aged 65 years or over was 703 million globally in 2019 and is projected to reach 1.5 billion by 2050 (1). Likewise, 176 million people (12.6%) aged 65 years or over in China, and the rising burden of non-communicable diseases (NCDs), poses severe challenges to the elder's quality of life (2) and the supply of HPC. Furthermore, living in environments affected by the coronavirus disease (COVID-19) pandemic and humanitarian crises are large-scale events that may pressure healthcare systems greatly. The World Health Organization (WHO) defines HPC as a crucial part of integrated care providing physical, mental, spiritual, and social care for older people and terminally ill patients by treating their discomfort, and is the basic skills of healthcare providers (3). Research shows that early delivery of HPC reduces unnecessary hospital admissions and services and optimizes patient health-related quality of life (4, 5). HPC should be available at all levels of care (6), but only 14% of patients currently have access to it worldwide. Policies for strengthening and expanding human resources are urgently needed to improve accessibility (7). The situation in China is more challenging due to the lack of professional HPC providers with positive behavioral intentions, poor public conception, and lack of knowledge regarding potential benefits (8). Also, targeted training is often limited, and HPC providers' knowledge and skills vary greatly, leading to unsatisfactory services (9). The WHO appeals to the local government and healthcare planners to estimate HPC demands and supplies using accurate data to combat the challenges (10). Therefore, there is an urgent need to evaluate the real-world situation of implementing HPC, particularly that of understanding the individual behavioral intentions that reflect whether healthcare providers are willing to engage in HPC. Exploration of the knowledge, attitude, and practice of hospice care (KAPHC) among doctors, nurses, and managers may yield useful information (11, 12), contribute to estimating individual behavioral intentions, and organize a productive service team to deliver HPC.

Existing studies mostly used traditional statistical methods to analyze the KAPHC among healthcare providers (13–15). There is abundant evidence suggesting that KAPHC is crucial for successful implementation of HPC (16–18). However, there is limited evidence about the use of combined real-world data and KAPHC to predict behavioral intentions. The main challenge of standardized surveys and many statistical models include limited data with unclear distribution. Thus, advanced machine learning and artificial intelligence enable the inclusion of large amounts of data based on correlational studies (19). Random Forest Classifier (RFC) may be more suitable for the analysis of behavioral intentions of HPC. One of the biggest advantages of RFC is that it can be used on data exhibiting highly unusual distribution by not making any distributional assumptions about underlying data structures. Additionally, it is a variation of bootstrap aggregating (bagging), which develops several hundred trees from the same dataset. The results are averaged from these trees, producing more accurate results to solve the problems of overfitting and being sensitive to small changes in the training data for a single Decision Tree (DT) (20). In addition, RFC allows the determination of feature importance measures for each parameter by measuring the effect of variable permutation on the model's accuracy (measured using out-of-bag error estimation) and node homogeneity (measured using the Gini index). In other words, it can tell each variable's importance in predicting the outcome (21). We applied the RFC to model impact and relate the prediction of individual behavioral intentions based on demographic parameters, career experience, and KAPHC to identify those that are willing to engage in HPC. RFC was introduced to predict behavioral intentions and review the model performance. In particular, this study aimed to detect correlative enablers and barriers in providing HPC.

## Methods

### Questionnaires

We conducted a cross-sectional study using the indigenized healthcare provider KAPHC scale, which proved to have good reliability (22). The scale mainly refers to the Palliative Care Quiz for Nursing by Ross et al. (23) and the Frommelt Attitudes Toward Care of the Dying Scale by Frommelt (24), Liu et al. (25) and Shimizu et al. (26). The three main components of the anonymous questionnaire were: demographic characteristics (gender and age group), career experience (location of the medical institution, type of medical institution), and measured data (score of knowledge, attitude, confidence, and practice).

### Data collection

Shanghai had received continuous support to improve HPC research and practices and achieve universal coverage of HPC among community health service centers (CHC) by 2020 (27). Therefore, we organized all the 228 medical institutions that registered an HPC department or were willing to provide HPC in Shanghai, to investigate healthcare providers' KAPHC and behavioral intentions from November 1 to December 31, 2019. Overall, 3709 HPC providers with working experience in HPC were invited to fill in the questionnaire independently, including doctors, nurses, managers, medical technicians, and others.

### Statistical methods

#### Descriptive statistics

The indicators were divided into two components: the target vector and the feature matrix. Behavioral intentions, a binary variable, were selected as the target vector, with the label (1/yes) suggesting positive behavioral intentions to work in HPC and the negative label (0/no) without behavioral intentions. The feature matrix includes demographic characteristics, career experience, and measure data of KAPHC. A higher score means better personal performance. Descriptive statistics for the feature matrix were based on full samples to analyze the situation of participation. Categorical variables were reported as frequency distribution and percentage, whereas continuous variables were reported as average values, standard deviations, median, and IQR (Inter Quartile Range). IBM SPSS Statistics for Windows, version 21.0 (IBM Corp., Armonk, NY, USA) software was used for the statistical analysis.

#### Logistic regression

Binary Logistic Regression (BLR) was performed to analyze the correlation between a dependent variable and independent variables to identify which variables can influence the willingness of healthcare providers to work in HPC. BLR could provide several key information points, namely, which explanatory variables show statistical significance with Wald's test, which variables are the risk or protective factors, and how important they are by odds ratio (OR). The goodness-of-fit is compared using the likelihood ratio test, the Hosmer–Lemeshow test, and Pearson's chi-squared test (28). BLR was used to select the relative potential features to control confounders and develop RFC. Moreover, the model fitting method was chosen as the stepwise way, and the probability for stepwise was reset such that entry was 0.1 and removal was 0.15 to avoid the type I error. Statistical analyses of the Logistic Regression model were performed using SPSS 21.0.

#### Random forest

**Figure 1:**
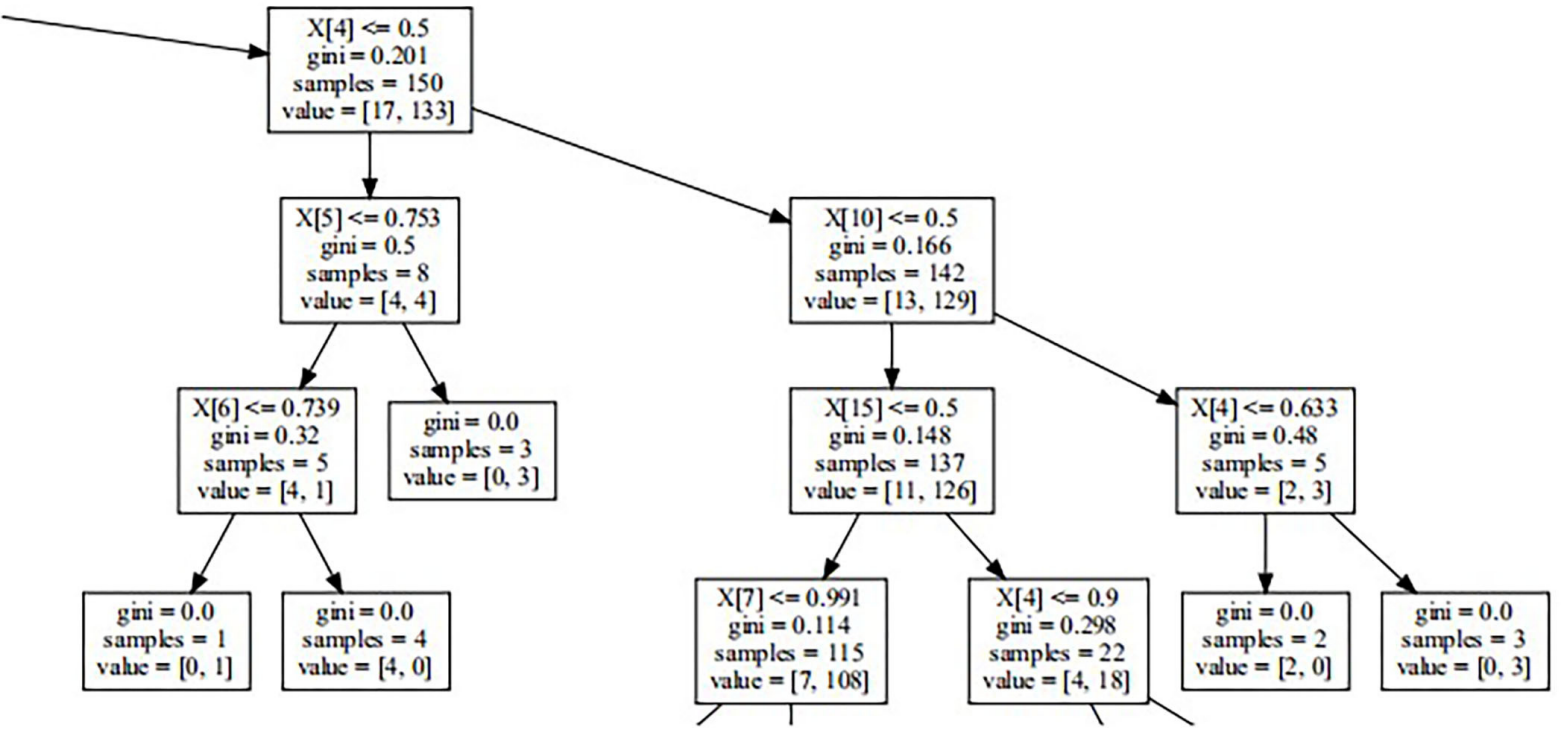
Part of decision tree. It reveals how decision tree works on the basis of Gini Impurity and other parameters. Random Forest is one of Ensemble Learnings developing from Decision Trees.

Random Forest (RF) lowers the variance of a single regression tree by averaging the prediction of multiple DTs. The DT is a non-parametric supervised learning method used for classification and regression analysis. The DT predicts the value of a target variable by learning simple decision rules inferred from the data features, which can be seen as a piecewise constant approximation (29). Each DT consists of edge conjunctions on a single variable greater than or less than some value. Each tree node divides the data into two subsets, thus making each subset more homogeneous (Figure 1). The terminal node (leaf) of the tree that a certain data point falls into determines the predictions by the majority selection of the training data (30). RF is a meta-estimator that fits several DT classifiers on various subsamples of the dataset and uses averaging to improve the predictive accuracy and control overfitting (31). It is a method by which several DTs (tree-like graphs of decisions and their possible consequences, including event outcomes) are built from the variable set. They divide participants into similar subgroups using the most significant variables. The prediction is subsequently generated using a “voting” scheme across all DTs. Gradient descent boosting also picks variables across DTs that best predict correct outcomes in their training sets (32).

In our study, the variable types of latent factors include nominal variables and skewed continuous variables. Related studies have proven the applicability of RF in assembling learning algorithms and machine learning methods with the advantages of no restrictions on variable conditions and higher accuracy, sensitivity, and specificity. In addition, RF could be utilized to predict continuous variables and obtain predictions without obvious deviations (33). Therefore, RFC (one of typical RF models) is considered a suitable prediction method for the data in this study. RFC was performed using the program Python, version 3.9.0 (Python Software Foundation., Beaverton, OR, USA) with packages including Sklearn, Numpy, and Pandas.

### Model development

We selected the potential features with statistical significance using BLR to develop our models. The data were normalized for having a zero mean and unit standard deviation, and randomly split into the 80% training dataset (*n* = 2,804) and the 20% test dataset (*n* = 701) (34). Considering the target vector was the binary variable, each feature was standardized between 0 and 1. Categorical features were encoded as the one-hot numeric array. This has created a binary column for each category and returned a sparse matrix or dense array (depending on the sparse parameter) (35). Model validation was performed using a 10-fold cross-validation approach on all the training data to avoid overfitting before RFC performance (36). Once preferable validation and reliability were confirmed, we trained the model on the complete training dataset, and the test dataset was used for the final validation. Meanwhile, RFC was compared with several widely used machine learning models to assess its advantage. Furthermore, the feature importance indicated the value of each feature in the RFC (37).

The predictive abilities of RFC were assessed using the following measures:

*Accuracy*: The proportion of the correctly classified samples. In multilabel classification, this function computes subset accuracy, meaning the set of labels predicted for a sample must match the corresponding set of labels in positive (38).

*Precision*: The ratio of “tp/(tp + fp)” where “tp” is the number of true positives and “fp” the number of false positives. The precision is intuitively the ability of the classifier not to label as positive a sample that is negative.

*Recall*: The ratio of “tp/(tp + fn)” where “tp” is the same as above and “fn” is the number of false negatives. The recall is intuitively the ability of the classifier to find all the positive samples.

*F1 score*: The weighted average of the precision and recall. The relative contribution of precision and recall to the F1 score are equal. F1 score = 2 ^*^ (precision ^*^ recall) / (precision + recall) (39).

*AUC*: The area under the Receiver Operating Characteristic curve (40).

*Average Precision (AP)*: The Precision-Recall curve shows the tradeoff between precision and recalls for different thresholds. A high area under the curve represents high recall and high precision. The AP summarizes a Precision-Recall curve as the weighted mean of precisions achieved at each threshold (41).

## Results

### Participants characteristics

Overall, 3,505 valid questionnaires were gathered with an effective response rate of 94.5%. There were 63.4% participants aged 30–50 years, the majority (75.7%) were women, and 76.3% were married. Additionally, 68.8% held a bachelor's degree (or above), and 16.1% had religious beliefs. For career experience, approximately half (49.6%) worked downtown and the others (50.45%) in the countryside. The majority were employed in CHC (64.5%), and most participants were doctors (36.7%) and nurses (39.25%). The government funded most medical institutions (82.7%). Among all the participants with certain working experience in HPC, only half (56.1%) still work in HPC positions at the time of the investigation. Details are given in Table 1.

**Table 1:** Characteristics of study participants (*n* = 3505).

**Characteristics**	***N***	**%**
**Gender**		
Male	850	24.3
Female	2,655	75.7
**Age group**		
≤ 30	1,022	29.2
30-−50	2,221	63.4
>50	262	7.5
**Educational background**		
Secondary specialized school (or below)	221	6.3
Junior college	873	24.9
Bachelor (or above)	2,411	68.8
**Nationality**		
Han nationality	3,347	95.5
Minority nationality	158	4.5
**Religious belief**		
Yes	563	16.1
No	2,942	83.9
**Marital status**		
Unmarried	681	19.4
Married	2,674	76.3
Divorce or widow	150	4.3
**Location of medical institution**		
Downtown	1,737	49.6
Countryside	1,768	50.4
**Type of medical institution**		
Nursing home and beadhouse	583	16.6
Hospital	663	18.9
Community health service center	2,259	64.5
**Fund or supply of medical institution**		
Government	2,900	82.7
Social or personal	605	17.3
**Occupation**		
Medical technician or others	346	9.9
Manager	500	14.3
Doctor	1,286	36.7
Nurse	1,373	39.2
**Profession title**		
None	259	7.4
Junior	1,215	34.7
Middle	1,626	46.4
Senior	405	11.6
**Experience of witness death**		
Yes	3,080	87.9
No	425	12.1
**Working in HPC now**		
Yes	1,967	56.1
No	1,538	43.9

The individual scores for attitude, practice, and requirement of training were more dispersed than those of knowledge and confidence. The average attitude was 91.7 points, and the standard deviation and IQR of attitude score were the highest among the five items. The average knowledge score was 8.9 points, with the lowest standard deviation and IQR. More details are reported in Table 2.

**Table 2:** Measure data of studied participants (*n* = 3,505).

**Item (full score)**	**$\bar{x}$ ± s**	**Median**	**IQR**	**n**
Attitude (125 points)	91.7 ± 12.6	92.0	17.0	3,505
Knowledge (15 points)	8.9 ± 2.7	9.0	4.0	3,505
Confidence (55 points)	41.0 ± 8.4	43.0	9.0	3,505
Practice (70 points)	50.6 ± 10.9	52.0	14.0	3,505
Requirement of training (30 points)	23.9 ± 7.0	27.0	11.0	3,505

### Feature selection

**Figure 2:**
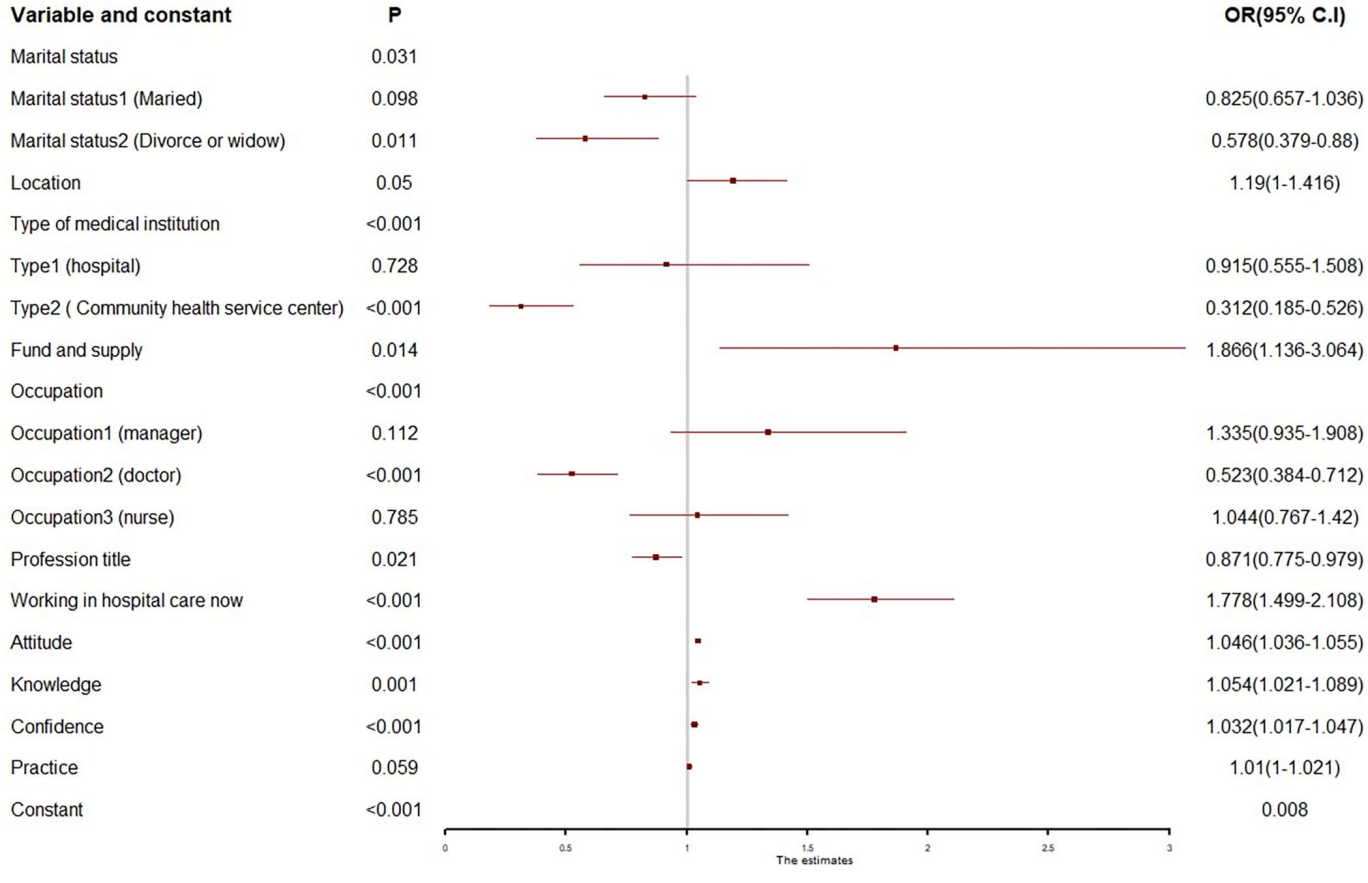
Forest plot of binary logistic regression. Only the variables with statistical significance were displayed in the plot.

Figure 2 presents the result of the last step in BLR. The Wald test indicated that marital status (*P* = 0.031), location of medical institution (*P* = 0.050), type of medical institution (*P* < 0.001), fund or supply of medical institution (*P* = 0.014), occupation (*P* < 0.001), profession title (*P* = 0.021), “Working in hospice care now” (*P* < 0.001), attitude (*P* < 0.001), knowledge (*P* = 0.001), confidence (*P* < 0.001), and practice (*P* = 0.059) showed statistical significance; these factors were selected as potential features fitting in RFC analysis. Meanwhile, it should be noted that KAPHC showed a positive impact on behavioral intentions, especially knowledge (OR = 1.054; 95% CI: 1.021–1.089), which is higher than others. Meanwhile, profession title, marital status 2 (Divorced or widow), medical institution 2 (CHCs), and occupation 2 (doctor) showed a negative impact on behavioral intentions, especially the medical institution of CHCs (OR = 0.312; 95% CI: 0.185–0.526).

### Cross-validation

Overall, most measures were over 0.75. The recall performance (average of 0.92) was the best, followed by AP and F1-score of about 0.85. Additionally, the average accuracy, precision, and AUC values were between 0.75 and 0.80. Details are given in Table 3.

**Table 3:** Result of cross-validation on training dataset (*n* = 2,804).

**Split**	**Accuracy**	**Precision**	**Recall**	**F1-score**	**AUC**	**AP**
1	0.75	0.80	0.91	0.86	0.72	0.87
2	0.77	0.80	0.95	0.87	0.77	0.88
3	0.77	0.79	0.91	0.87	0.74	0.88
4	0.75	0.80	0.90	0.83	0.71	0.87
5	0.75	0.80	0.91	0.85	0.71	0.85
6	0.76	0.78	0.92	0.85	0.75	0.87
7	0.78	0.79	0.91	0.84	0.75	0.87
8	0.79	0.82	0.91	0.85	0.75	0.87
9	0.74	0.78	0.91	0.84	0.76	0.88
10	0.76	0.78	0.93	0.86	0.75	0.89
Avg.	0.76	0.79	0.92	0.85	0.74	0.87

### Model testing

**Figure 3:**
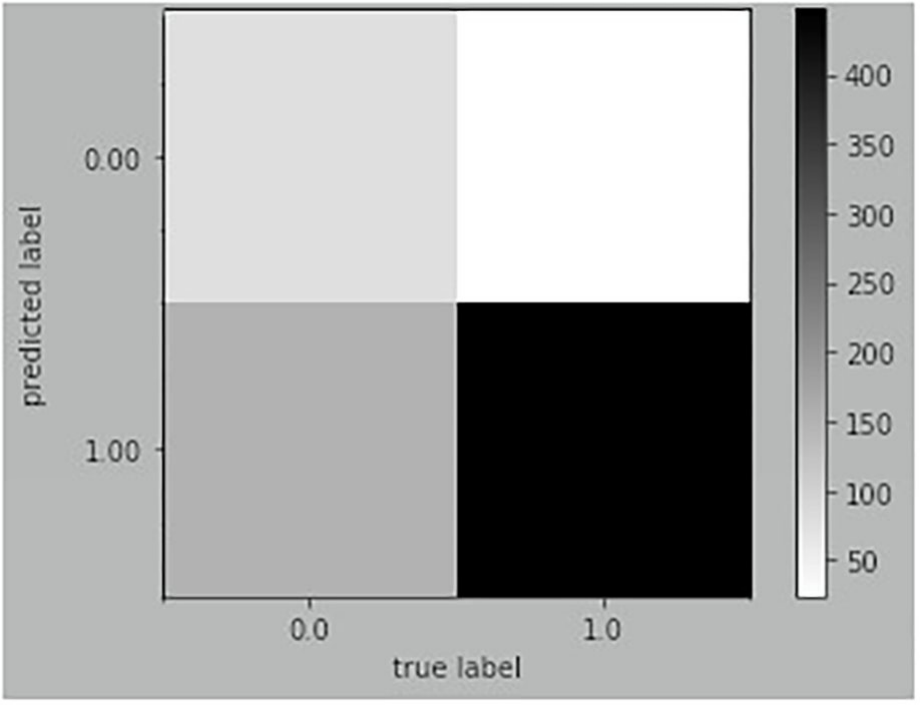
Confusion matrix of RFC on testing dataset.

**Figure 4:**
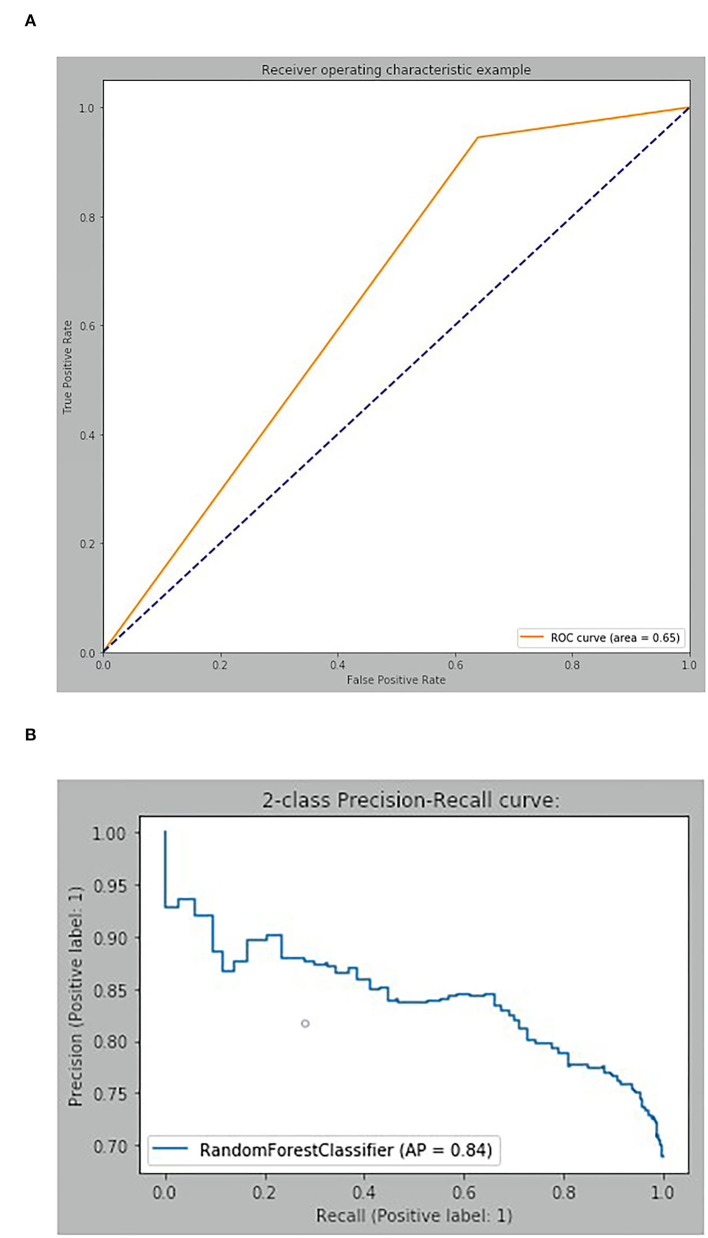
Discriminatory ability of Random Forest Classifier. **(A)** Receiver operating characteristic curve. **(B)** Two-class Precision-Recall curve.

RFC was compared with three widely used machine learning models, including DT, K-Nearest Neighbor (KNN), and Support Vector Machine (SVM), to prove its superiority. Generally, RFC performed better for most measures, while the recall of SVM was higher than that of RFC. More details are given in Table 4. The result of RFC prediction on the testing dataset is illustrated in a confusion matrix (Figure 3). In the testing dataset, 592 observations were classified as a positive label and 109 as negative. The true positive and false negative rates were 0.94 and 0.64, respectively. The recall of the RFC model was 0.94, and the F1-score was 0.84; the accuracy was similar to the precision (0.75). Also, the AUC of the RFC model was 0.65. The AP of the model was 0.84, which is similar to the average value of cross-validation. More details are shown in Figure 4.

**Table 4:** Model performance on testing dataset.

**Model**	**Accuracy**	**Precision**	**Recall**	**F1-score**	**AUC**
RFC	0.75	0.75	0.94	0.84	0.65
DT	0.66	0.73	0.77	0.75	0.59
KNN	0.73	0.74	0.92	0.82	0.62
SVM	0.73	0.72	0.99	0.83	0.59

### Feature importance

**Figure 5:**
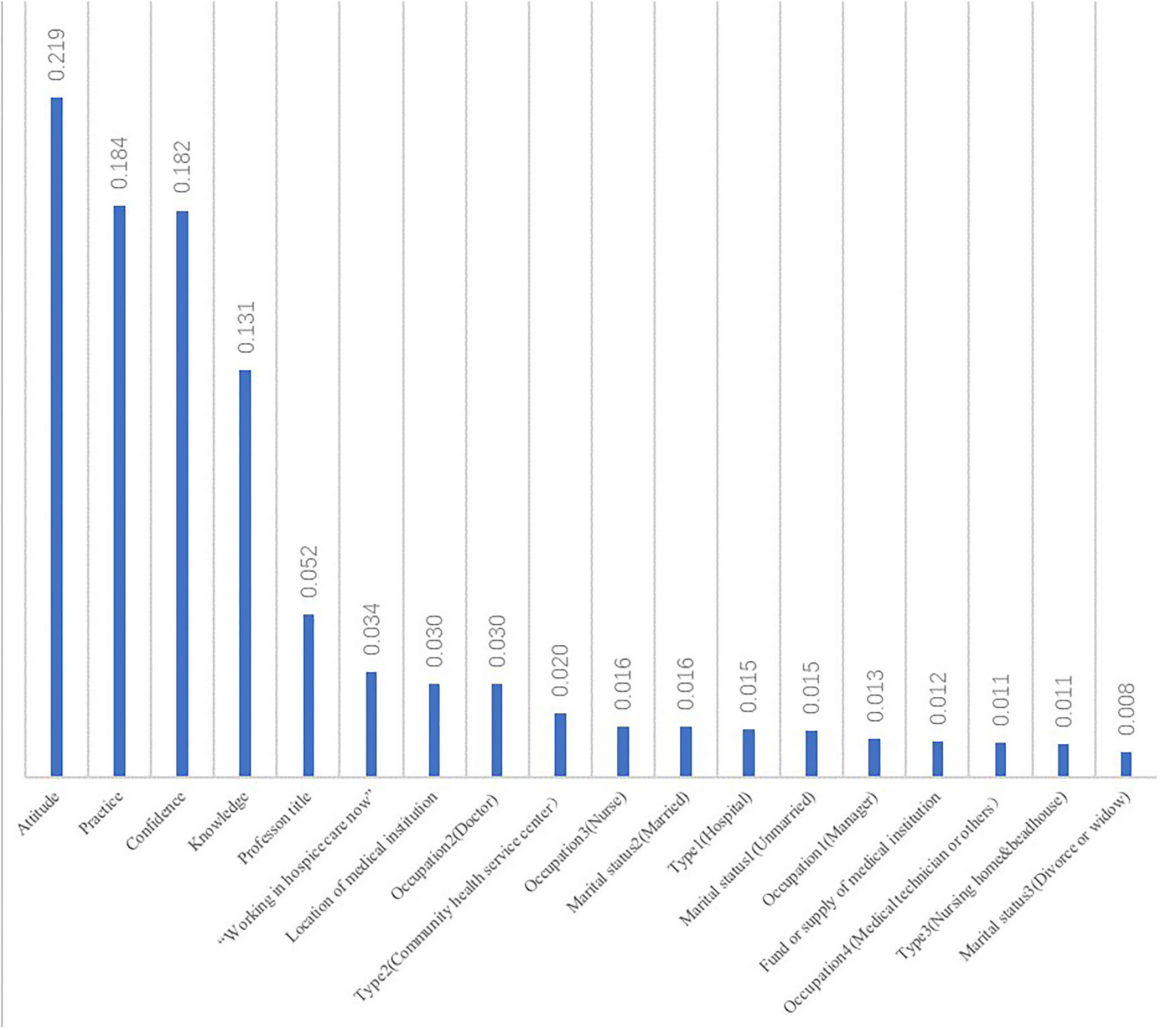
Feature importance. It shows how important the value was in the model.

The feature importance was computed as the (normalized) total reduction of the criterion brought by that feature, also known as the Gini importance (42). It was indicated that features from measure data were much more important than others. Attitude (0.219) was the most significant factor, followed by practice (0.1840), confidence (0.182), and knowledge (0.131). Feature importance of profession title, “Working in hospice care now,” location of the medical institution, and occupation 2 (Doctor) were 0.030 and over. All details are given in Figure 5.

## Discussion

### Model performance

The result of RFC showed high sensitivity, suggesting that a majority of the samples were correctly classified into a positive label. However, the AUC (0.65) was still far from that of a perfect classification. It is considered that the class imbalance of the target variable caused the capacity of the discrimination to be poor, suggesting that we could not evaluate our classification models completely based on the accuracy and AUC. The Precision-Recall curve is a widely used measure of prediction for imbalanced classes (43). The AP was 0.84, which satisfied our expectations. Meanwhile, according to the study target of developing a productive HPC team, we paid more attention to whether the model contributed to identifying healthcare providers with positive behavioral intentions, focusing on the recall and the true positive rate of the model. Finally, the RFC was considered to have practical application value and could be a reasonable method for estimating individual behavioral intentions of HPC, organizing a productive team, and evaluating the ability to implement HPC.

### Attitude was the most significant enabler

According to the outcome of RFC and BLR, attitude was the most significant enabler. A positive attitude was a crucial facilitator to improving the quality of HPC services and important for building teamwork and interpersonal relationship (44, 45). It was remarkable that healthcare providers who were divorced or widowed showed a negative attitude to work in HPC, owing to painful experiences that trigger negative emotions, nervousness, and other barriers. The correlative survey also highlighted that attitude toward caring for dying patients was mainly influenced by death anxiety of the healthcare provider (46). One would develop negative emotions, and death avoidance was the typical performance (47). Nurses, being the main providers of HPC, were the most probable participants experiencing death anxiety with a direct effect on their attitude toward caring for the dying (48). Further, death anxiety was also induced by high levels of burnout and occupational stress among healthcare providers. They may struggle to realize work value and career satisfaction (49). Therefore, healthcare providers must develop a positive attitude toward death to optimize the quality of HPC.

In our further analysis, aside from its own influence, attitude was associated with other features that affect behavioral intentions (50). Confidence was closely correlated with attitude. Healthcare providers with negative attitudes had insufficient confidence and rejected or escaped from caring for dying patients, which constituted a serious barrier to improving the quality of HPC (48). Also, it was noteworthy that healthcare providers still working in HPC showed a positive attitude. This shows that long-term practice or constant work in HPC could help service providers develop positive attitudes, which might release their death anxiety and discomfort for the death or dying patients (51).

### Practice experience enhances the development of behavioral intentions

BLR and RFC provided similar evidence suggesting that practice experience would enhance the development of behavioral intentions. It has been illustrated that healthcare providers at CHCs show a lack of behavioral intentions, partly due to assigned work tasks by the government instead of by active participation, without sufficient financial support and an effective incentive mechanism. Worse still, in many CHCs, there are rare opportunities to practice since the public are unaware of HPC (52). Furthermore, confidence (self-efficacy) toward HPC would be influenced by the practice experience. Therefore, providing opportunities for practices could improve self-efficacy in providing HPC, suggesting the need for sustained government funding and long-term incentives, such as a reasonable payment system, to inspire the providers' enthusiasm now and in the long-term, and to strengthen public education of HPC. In addition, there should be a standardized training design, communication platform, or academic salon to encourage healthcare providers to discuss practice, personal involvement, and further research on HPC (53).

### Knowledge is an undeniable facilitator

BLR and RFC highlighted that knowledge was an undeniable facilitator of behavioral intentions. The impact of knowledge includes two aspects: whether healthcare providers understand the concept of HPC and whether they have a good knowledge of the essential skills and apply them to HPC practice. For the former, we noticed that doctors showed negative behavioral intentions compared with other professionals. Most doctors would rarely consider HPC when cancer patients first visited; however, they agreed with the value of HPC (54). Doctors aim to cure patients or recover them from diseases to maintain life; however, some doctors still consider that HPC implies abandoning treatment. They tend to struggle to maintain life-prolonging treatment and gain poor satisfaction from HPC services (55). Therefore, it is urgent to disseminate the concept and benefits of HPC among healthcare providers and the public to help establish the concept of integrated life-cycle care and HPC for terminal patients.

For the latter, we found that healthcare providers working in the countryside showed negative behavioral intentions due to a lack of knowledge and essential skills of HPC. As previously reported, most providers lack the professional training, skills, and principles in HPC practice, particularly pain control, symptom management, and psychological care (56). Also, one-third of physicians had little knowledge of the HPC guidelines, and two-thirds did not know the Advance Care Planning manual (57). This might cause insufficient confidence, poor behavioral intentions, and impede practices and implementation of HPC.

## Limitations

RFC outperformed other machine learning models and avoided the overfitting phenomenon; however, it is not a perfect classification. Therefore, we will keep working to develop more excellent methods. Additionally, evidence from Shanghai could not represent the situation of other provinces in China. Therefore, we will try to expand our samples to other areas to get stable evidence.

## Conclusion

HPC has become a topic of interest owing to the rising burden of NCDs, population aging, and the COVID-19 pandemic. However, due to several barriers to implementing HPC, most patients cannot access the needed professional services. Therefore, it is significant to understand the real-world situation at the initial stage of the HPC movement in China, particularly the individual behavioral intentions to improve the accessibility of HPC. Our study was designed to predict the behavioral intentions of HPC providers using RF, which presented a first attempt in this area to automatically identify the relationship between behavioral intentions and other parameters of KAPHC. RFC showed high sensitivity, which suggested a well-trained RFC could help to estimate individual behavioral intentions to organize a productive team. Moreover, attitude is considered the most significant facilitator, implying the most efficient approach to encourage behavioral intentions. Reasonable HPC knowledge and practices in caring for terminal patients are of potential interest and stimulate the behavioral intentions of healthcare providers. Ultimately, the study has provided evidence that the government and HPC organizations should scale up standardized training, sustained financial support, and long-term incentives to attract providers to engage actively and enthusiastically in HPC. This will ensure its long-term sustainable development in China.

## Data availability statement

The raw data supporting the conclusions of this article will be made available by the authors, without undue reservation.

## Ethics statement

This study was approved by the Ethics Committees of Shanghai Ninth People's Hospital (Ref: SH9H-2021-T11-1). Written informed consent from the participants was not required to participate in this study in accordance with the national legislation and the institutional requirements.

## Author contributions

TC contributed to the original draft's conceptualization, formal analysis, and writing. YX contributed to the investigation and data curation. HZ contributed to visualization. XT contributed to the investigation and data curation. LJ contributed to writing—review and editing, supervision, and project administration. All authors contributed to the article and approved the submitted version.

## Funding

This work was supported by grants from the Nature Science Foundation of Shanghai (No. 22ZR1461400), Humanities and Social Science Research Planning Fund of the Ministry of Education (No. 20YJAZH045), Philosophy and Social Science Planning Project of Shanghai (No. 2019BGL032), and Pujiang Talent Program of Shanghai (No. 2019PJC099).

## Conflict of interest

The authors declare that the research was conducted in the absence of any commercial or financial relationships that could be construed as a potential conflict of interest.

## Publisher's note

All claims expressed in this article are solely those of the authors and do not necessarily represent those of their affiliated organizations, or those of the publisher, the editors and the reviewers. Any product that may be evaluated in this article, or claim that may be made by its manufacturer, is not guaranteed or endorsed by the publisher.
